# Sequestration of latent TGF-β binding protein 1 into CADASIL-related Notch3-ECD deposits

**DOI:** 10.1186/s40478-014-0096-8

**Published:** 2014-08-13

**Authors:** Jessica Kast, Patrizia Hanecker, Nathalie Beaufort, Armin Giese, Anne Joutel, Martin Dichgans, Christian Opherk, Christof Haffner

**Affiliations:** Institute for Stroke and Dementia Research, Klinikum der Universität München, Ludwig-Maximilians University Munich, Max-Lebsche-Platz 30, 81377 Munich, Germany; Munich Center for Systems Neurology (SyNergy), Schillerstr. 44, 80336 Munich, Germany; Center for Neuropathology and Prion Research, Ludwig-Maximilians University Munich, Feodor-Lynen-Str. 23, 81377 Munich, Germany; INSERM, U1161, F-75010 Paris, France; Univ Paris Diderot, Sorbonne Paris Cité, 10 av de Verdun, UMRS 1161, F-75010 Paris, France

**Keywords:** Latent TGF-β binding protein 1 (LTBP-1), Small vessel disease, CADASIL, TGF-β signaling, Granular osmiophilic material (GOM), Extracellular matrix (ECM)

## Abstract

**Introduction:**

Cerebral autosomal dominant arteriopathy with subcortical infarcts and leukoencephalopathy (CADASIL) represents the most common hereditary form of cerebral small vessel disease characterized by early-onset stroke and premature dementia. It is caused by mutations in the transmembrane receptor Notch3, which promote the aggregation and accumulation of the Notch3 extracellular domain (Notch3-ECD) within blood vessel walls. This process is believed to mediate the abnormal recruitment and dysregulation of additional factors including extracellular matrix (ECM) proteins resulting in brain vessel dysfunction. Based on recent evidence indicating a role for the transforming growth factor-β (TGF-β) pathway in sporadic and familial small vessel disease we studied fibronectin, fibrillin-1 and latent TGF-β binding protein 1 (LTBP-1), three ECM constituents involved in the regulation of TGF-β bioavailability, in *post-mortem* brain tissue from CADASIL patients and control subjects.

**Results:**

Fibronectin and fibrillin-1 were found to be enriched in CADASIL vessels without co-localizing with Notch3-ECD deposits, likely as a result of fibrotic processes secondary to aggregate formation. In contrast, LTBP-1 showed both an accumulation and a striking co-localization with Notch3-ECD deposits suggesting specific recruitment into aggregates. We also detected increased levels of the TGF-β prodomain (also known as latency-associated peptide, LAP) indicating dysregulation of the TGF-β pathway in CADASIL development. *In vitro* analyses revealed a direct interaction between LTBP-1 and Notch3-ECD and demonstrated a specific co-aggregation of LTBP-1 with mutant Notch3.

**Conclusion:**

We propose LTBP-1 as a novel component of Notch3-ECD deposits and suggest its involvement in pathological processes triggered by Notch3-ECD aggregation.

**Electronic supplementary material:**

The online version of this article (doi:10.1186/s40478-014-0096-8) contains supplementary material, which is available to authorized users.

## Introduction

Cerebral small vessel disease (SVD), a major cause of stroke and vascular dementia, represents the most prevalent neurological disorder in aging societies and thus a considerable health care problem [1,2]. Monogenic forms of SVD such as cerebral autosomal dominant arteriopathy with subcortical infarcts and leukoencephalopathy (CADASIL) are considered valuable model conditions contributing to our understanding of SVD pathomechanisms [3,4]. CADASIL vessel pathology shows considerable overlap with sporadic SVD including progressive thickening of the tunica intima and tunica adventitia as well as narrowing of the vascular lumen. These alterations are likely a result of fibrotic events involving increased production of extracellular matrix (ECM) proteins such as collagens [5,6]. They are observed primarily in small pial and penetrating arteries, arterioles and capillaries of the brain resulting in reduced cerebral blood flow and white matter damage [7,8].

The gene mutated in CADASIL, *NOTCH3,* encodes a transmembrane receptor required for arterial differentiation and maturation of vascular smooth muscle cells in small arteries [9]. Pathogenic mutations predominantly affect cysteine residues within individual epidermal growth factor (EGF)-like repeats of the Notch3 extracellular domain (Notch3-ECD) [10,11] leading to Notch3-ECD multimerization and accumulation in the tunica media of vessel walls [12]. Notch3-ECD aggregates coincide with large electron-dense deposits known as granular osmiophilic material (GOM), an invariant feature of CADASIL-affected vessels [13-15]. The appearance of Notch3-ECD aggregates prior to neurological symptoms in both patients [16,17] and mouse models [18-20] suggest that they represent an early manifestation causative for disease development.

The molecular mechanisms underlying Notch3-ECD deposit formation and the pathological events leading to vessel dysfunction are incompletely understood. A variety of studies using cultured cells or mouse models [19,21-23] have failed to detect alterations in signaling capacity of CADASIL-mutant Notch3, although contradictory results have been reported [24]. Moreover, recently identified patients with hypomorphic *NOTCH3* alleles do not show signs of CADASIL [25]. Thus, novel pathogenic roles for mutant Notch3 rather than compromised Notch3 function have been proposed as the primary determinant of the disease [9].

Using scanning for intensely fluorescent targets (SIFT), a confocal technique developed for monitoring protein multimerization in solution [26], we have recently recapitulated the Notch3 aggregation process *in vitro* and demonstrated its facilitation by CADASIL mutations [27,28]. Moreover, we observed co-aggregation of the matricellular protein thrombospondin-2 [28], a known Notch3 interactor and regulator of ECM assembly [29], providing experimental evidence for a pathological co-aggregation mechanism. This is supported by recent results obtained from CADASIL brain material enriched for Notch3-ECD deposits by sequential fractionation [30]. Using a mass spectrometry detection approach, a variety of proteins were found to co-fractionate with Notch3-ECD and for two of them, TIMP-3 and vitronectin, disease-related roles were proposed. Notch3-ECD aggregation might thus represent the initiating event of a continuative process involving the recruitment and sequestration of proteins with important roles in normal vessel function.

The dysregulation of the transforming growth factor-β (TGF-β) signaling pathway, a key regulator of fibrotic events in various organs including the vasculature [31], has been suggested to contribute to SVD pathogenesis [32]. Moreover, increased TGF-β activity has recently been reported in cerebral autosomal recessive arteriopathy with subcortical infarcts and leukoencephalopathy (CARASIL), a recessively inherited SVD syndrome related to CADASIL [33,34]. A specific role of TGF-β in CADASIL can be inferred from the fact that fibronectin, fibrillin-1 and members of the latent TGF-β binding protein (LTBP) family, important ECM components with a role in TGF-β bioactivation [35], were among the factors identified in the proteomic study on CADASIL brains [30]. We now extend this finding using immunohistological approaches on *post-mortem* brain material and report a dramatic enrichment of fibronectin, fibrillin-1 and LTBP-1 in CADASIL vessels. While fibronectin and fibrillin-1 show an immunohistological staining pattern different from Notch3-ECD suggesting accumulation due to fibrotic events secondary to Notch3-ECD aggregation, a striking co-localization with Notch3-ECD deposits was observed for LTBP-1 indicating a role in Notch3-ECD-mediated toxicity. This finding is further supported by analyses demonstrating a direct interaction of LTBP-1 with Notch3-ECD and a co-aggregation with mutant Notch3 *in vitro*.

## Materials and methods

### Brain samples and vessel isolation

Frozen brain samples of five genetically confirmed CADASIL patients (CAD 1–5) and four age- and sex-matched controls with no known cerebrovascular disorders (Ctrl 1–4) as well as paraffin sections of cases 1–3 and controls 1–3, all from the frontal subcortex (see Table 1), were provided by the Brain-Net Biobank (Ludwig-Maximilians-University Munich) and the CADASIL brain bank (INSERM, Paris) [30]. Their distribution and analysis occurred according to the guidelines of the local ethical committees. Due to limited amounts of obtained material, microvessel isolation could only be performed from CAD 2–5 and Ctrl 1–4 samples. Following a modified version of a previously described protocol [30], 100 mg brain tissue were homogenized in 15 mL cold Minimum Essential Medium (MEM) (Gibco, Life Technologies) in a glass tissue grinder (Wheaton), homogenates adjusted to a final concentration of 15% Ficoll and centrifuged at 6000 g and 4°C for 20 min. The pelleted vessels were resuspended in phosphate-buffered saline (PBS)/1% BSA and transferred onto a 40-μm nylon mesh (Corning Life Sciences). After extensive washing with PBS, isolated vessels were collected in a 50-mL tube and pelleted for 5 min at 3000 g. The purity of the vessels was verified using a phase-contrast microscope.

**Table 1:** **Overview of autopsy cases**

**Description**	**Sex**	**Age**	***NOTCH3*** **mutation**	**Applications**
CAD 1	M	64	R110C	Histological stainings
CAD 2	F	66	D239_D253del	Histological stainings, Western Blot
CAD 3	M	68	C144S	Histological stainings, Western Blot
CAD 4	F	60	R153C	Western Blot
CAD 5	F	70	C1261R	Western Blot
Ctrl 1	M	61	-	Histological stainings, Western Blot
Ctrl 2	F	55	-	Histological stainings, Western Blot
Ctrl 3	F	60	-	Histological stainings, Western Blot
Ctrl 4	F	73	-	Western Blot

CAD: CADASIL patient; Ctrl: control patient. Applications were limited by sample amount and type (frozen or paraffin, see Material and methods).

### Immunohistological analysis

For immunofluorescence analysis of CAD 1–3 and Ctrl 1–3 samples, frozen 10-μm tissue sections were thawed to room temperature and fixed for 15 min in 4% paraformaldehyde (PFA) (Morphisto), blocked with PBS/5% donkey serum (Jackson ImmunoResearch) for 40 min and probed with primary antibodies in PBS/1% donkey serum overnight at 4°C. For detection, Alexa Flour 488-, Cy3- or rhodamine-coupled secondary antibodies (Jackson ImmunoResearch, 1:200) were applied for 1 h at room temperature. After mounting in ProLong Gold antifade reagent (Life Technologies), slides were examined using an inverse (Axiovert 200 M, Zeiss equipped with AxioCam and AxioVision software) or confocal (TCS SP5, Leica equipped with LAS LF software) microscope.

For immunohistochemical analysis of CAD 1–3 and Ctrl 1–3 samples, 8-μm paraffin sections were deparaffinized, rehydrated and antigens unmasked by boiling in 10 mM citrate buffer, pH 6.0 for 30 min. Blocking was performed for 1 h in PBS/5% donkey serum and primary antibodies incubated in PBS/1% donkey serum overnight at 4°C. After three washes in PBS and blocking of endogenous peroxidases with 0.3% hydrogen peroxide for 15 min, appropriate biotinylated secondary antibodies (DAKO or Vector laboratories, 1:200) were applied for 1 h at room temperature. Development was performed with avidin/biotin-horseradish peroxidase (HRP) complex (Vectastain ABC-HRP kit, Vector Laboratories) and 3-amino-9-ethylcarbazole (AEC Peroxidase substrate Kit, Vector Laboratories). To provide cytological detail, sections were counterstained with Mayer’s hematoxylin Solution (Sigma) for 1 min and rinsed in tap water for 10 min prior to mounting with ProLong Gold. Sections were examined using the Axio Imager M2 microscope (Zeiss).

The following primary antibodies were used: goat polyclonal anti-collagen type IV (Southern Biotech, 1:500), rabbit polyclonal anti-fibrillin-1 (kind gift of Lynn Sakai, Shriners Research Center, Portland [36], 1:100), mouse monoclonal anti-LTBP-1 (MAB388, R&D Systems, 1:50), mouse monoclonal anti-fibronectin (F3648 Sigma, 1:300), goat polyclonal anti-LAP (AF-246, R&D Systems, 1:50) or rat monoclonal anti-Notch3-ECD (clone 2G8, raised against the peptide NH_2_-RSFPGSPPGASNASC-COOH, kind gift of Elisabeth Kremmer, Helmholtz Center, Munich, 1:10).

### Sequential protein extraction

Protein extraction from purified brain vessels was performed by modifying a previously published protocol [30]. Vessels were homogenized in 50 mM Tris–HCl, pH 7.5 using a TissueLyzer (Qiagen) with 5-mm beads for 3 min at 50 Hz. After clearance by centrifugation (5 min, 16,300 g), the Tris fraction was collected and the pellet resuspended in 50 mM Tris–HCl, pH 7.5, 1% SDS for 30 min on ice. Centrifugation for 30 min at 16,300 g yielded the SDS fraction and a pellet, which was resuspended in 50 mM Tris–HCl, pH 7.5, 1% SDS, 5% β-mercaptoethanol (β-ME) for 30 min on ice while passing through a 29-gauge needle 5 times. The β-ME fraction was obtained by centrifugation for 30 min at 16,300 g. Protease inhibitors (complete Mini EDTA-free, Roche) were used throughout the extraction procedure.

### Plasmids

The short human isoform of LTBP-1 (LTBP-1S) consisting of 1395 amino acids (aa) was used throughout the study. Constructs encoding full-length LTBP-1S and an N-terminally truncated variant encoding aa 528–1395 with a carboxy-terminal hemagglutinin (HA) tag (LTBP-1∆N_HA) [37] were kindly provided by Jorma Keski-Oja (University of Helsinki, Finland). Full-length LTBP-1S was subcloned into the pEAK-12 vector (provided by Stefan Lichtenthaler, DZNE, Munich) to harbour the CD5 signal peptide, an N-terminal HA tag and a C-terminal V5-His tag. LTBP-1∆N containing a C-terminal V5-His tag (LTBP-1∆N_V5) was generated from the full-length clone and LTBP-1∆N_HA by restriction fragment cloning. A C-terminally truncated LTBP-1 variant encoding aa 1–689 and a C-terminal V5-His tag (LTBP-1∆C-V5) in the pTT5 vector [28] was constructed from full-length LTBP-1 by restriction fragment cloning. The N3EGF1-5 wild-type (WT) and N3EGF1-5 C183R constructs used previously [28] were modified to contain a C-terminal Halo tag (Promega).

### Cell transfection

Human embryonic kidney (HEK) 293T cells were grown in Dulbecco’s Modified Eagle‘s Medium (DMEM)-GlutaMAX supplemented with 10% fetal calf serum and Penicillin/Streptomycin (all from Gibco, Life Technologies). Small-scale transfections were carried out in 24-well plates using 250 ng plasmid and Fugene (Roche) according to the manufacturer’s protocol and serum-free conditioned supernatant was collected after 48 h and cleared by centrifugation. The large-scale transfection procedure used for protein purification from HEK293E cells has been described earlier [28].

### Protein purification

Purification of His-tagged proteins (LTBP-1ΔC_V5 and LTBP-1ΔN_V5) was performed analogous to a protocol previously described [28]. Purification of N3-EGF1-5 using the Halo System (Promega) will be described elsewhere. Eluted fractions were analyzed by SDS-PAGE followed by silver staining to verify the protein purity. Protein concentrations were measured using the Qubit protein assay (Life Technologies) or BCA Protein Assay Kit (Pierce, Thermo Scientific) according to the manufacturer’s instructions.

### SDS-PAGE, Western blot and silver staining

Proteins were separated on polyacrylamide gels (Minigel system, BioRad) and either transferred onto Immobilon-P Membrane (Millipore) using the semi-dry system (Trans-Blot, BioRad) or processed for silver staining (Roti-Black Kit, Roth). For Western blotting, membranes were blocked with Tris-buffered saline (TBS)-0.05% Tween/4% skim milk for 1 h and probed with the following primary antibodies in TBS-0.05% Tween/4% skim milk overnight: mouse monoclonal anti-V5 (R960, Invitrogen, 1:1000), rat monoclonal anti-Notch3 ECD (clone 3G6, [28] 1:20), goat polyclonal anti-LAP (AF-246-NA, R&D Systems, 1:500), mouse monoclonal anti-LTBP-1 (MAB388, R&D Systems, 1:500) and mouse monoclonal anti-β-Tubulin (clone TUB 2.1, Sigma, 1:1000). Detection was carried out using HRP-coupled secondary antibodies (DAKO), chemiluminescence development (Immobilon Western HRP Substrate, Millipore) and the Fusion FX7 imaging system (Vilber Lourmat).

### Solid-phase binding assay

1 μg recombinant human Notch3 encompassing aa 40–467 (N3EGF1-11-Fc) and IgG_1_-Fc (both R&D Systems) were dissolved in PBS and coated onto Maxisorp 96-well plates (Nunc) overnight at 4°C. After blocking with PBS/1% BSA for 1 h, 50 μL conditioned supernatant from HEK293T cells transfected with LTBP-1, LTBP-1ΔC_V5, LTBP-1ΔN_HA or 100 ng purified LTBP-1ΔC_V5 in PBS were allowed to bind for 1 h at room temperature. Supernatant of mock-transfected cells or PBS was used as a negative control. After washing, detection was performed for 1 h using the following antibodies: mouse monoclonal anti-V5 (R960, Invitrogen, 1:500) for LTBP-1 and LTBP-1∆C_V5; mouse monoclonal anti-LTBP-1 (MAB388, R&D Systems, 1:500) for LTBP-1∆N_HA. Following incubation with anti-mouse-HRP (DAKO, 1:1000) for 1 h, immune complexes were visualized by TMB Microwell Peroxidase Substrate Kit (KPL) according to the manufacturer’s protocol. For Western Blotting, bound proteins were solubilized in Laemmli buffer.

### Scanning for intensely fluorescent targets (SIFT)

The fluorescent labeling of proteins, incubation for protein aggregation and confocal single-particle analysis were essentially performed as previously described [28]. Prior to the assay, labeled proteins were centrifuged at 100,000 g for 1 h at 4°C to remove preformed aggregates. Protein concentrations used for aggregation experiments were 64 nM (LTBP-1ΔC_V5 and LTBP-1ΔN_V5) and 160 nM (N3EGF1-5 WT and N3EGF1-5 R183C). SIFT measurements were performed after 24 h on an Insight II Reader (Evotec-Technologies).

### Statistical analysis

Data are expressed as mean and standard error of the mean (SEM) of the indicated number of experiments. Statistical analysis was performed using the Sigma Plot 12.5 software and the Mann–Whitney Test.

## Results

### Fibronectin and fibrillin-1 do not co-localize with Notch3-ECD deposits

**Figure 1:**
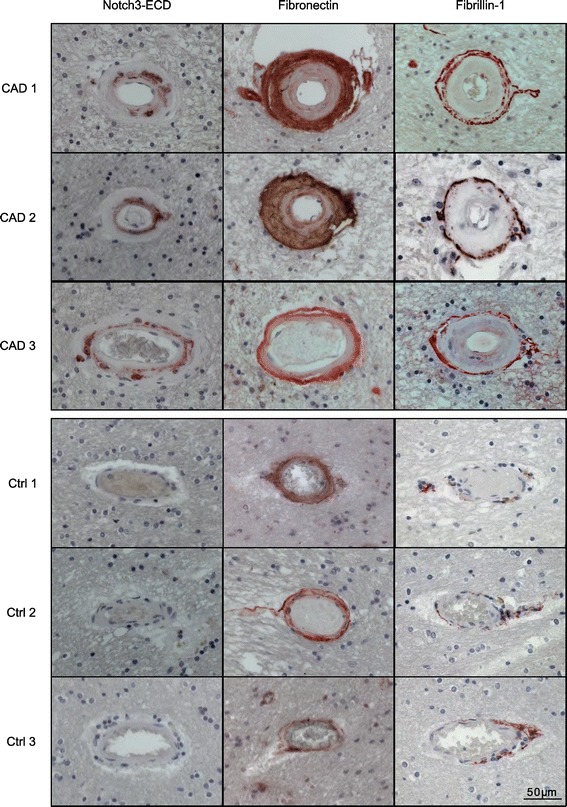
**Fibronectin and fibrillin-1 accumulate in CADASIL brain vessels.** Paraffin-embedded brain sections of three CADASIL patients and three healthy controls were stained for Notch3-ECD, fibronectin and fibrillin-1, counterstained with hematoxylin (blue) and analyzed by bright-field microscopy. (CAD: CADASIL patient; Ctrl: control).

**Figure 2:**
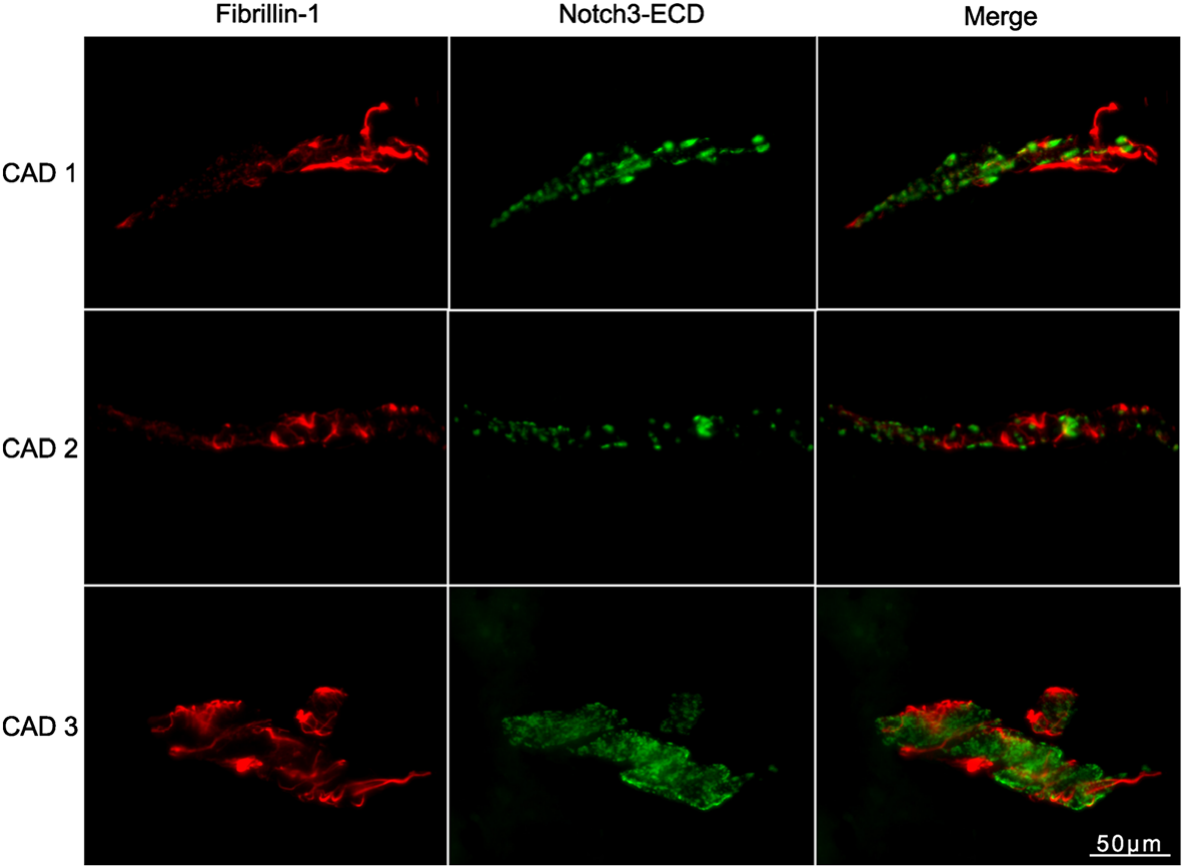
**Fibrillin-1 and Notch3-ECD do not co-localize.** Frozen brain sections from three CADASIL patients were co-stained for fibrillin-1 and Notch3-ECD and analyzed by fluorescence microscopy. (CAD: CADASIL patient).

To study potential molecular mechanisms involved in mediating the toxicity of Notch3-ECD deposits *post-mortem* brain material from five CADASIL patients and four age- and sex-matched controls was analyzed by different experimental approaches (Table 1). Immunohistochemical staining of Notch3-ECD on paraffin-embedded sections confirmed elevated immunoreactivity in the tunica media of patient arterioles demonstrating the presence of Notch3-ECD deposits (Figure 1). Next, fibronectin, fibrillin-1 and latent transforming growth factor-β (TGF-β) binding protein 1 (LTBP-1), three ECM proteins with a known role in TGF-β bioactivation [35], were analyzed. Fibronectin was detected in moderate amounts in all layers of control vessels, but showed a dramatic increase in the thickened tunica adventitia and intima of CADASIL vessels (Figure 1). Similarly, staining of fibrillin-1 was weak in control vessels showing a fibrillar structure within the tunica adventitia, but was markedly elevated in CADASIL vessels resulting in the continuous labeling of a thin layer at the perimeter of the tunica adventitia (Figure 1). Thus, fibronectin as well as fibrillin-1 accumulate in CADASIL vessels, but their localization appears to differ from Notch3-ECD. The lack of spatial overlap was further demonstrated by immunofluorescence co-staining of frozen patient brain sections for fibrillin-1 and Notch3-ECD (a similar analysis for fibronectin was not possible due to the lack of a suitable antibody). While Notch3-ECD was exclusively detected as granular deposits typical for CADASIL vessels [12], fibrillin-1 immunoreactivity again showed a fibrous pattern not overlapping with Notch3-ECD staining (Figure 2). These data suggested that fibrillin-1 and fibronectin accumulation in CADASIL vessels is not a result of recruitment into Notch3-ECD deposits, but rather a consequence of fibrotic processes. We, therefore, excluded both proteins as direct participants in Notch3-ECD aggregation.

### LTBP-1 is recruited to Notch3-ECD deposits

**Figure 3:**
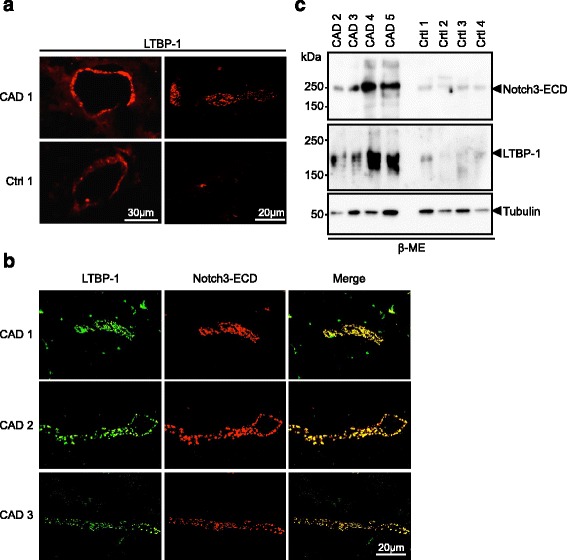
**LTBP-1 accumulates in CADASIL brain vessels and co-localizes with Notch3-ECD aggregates. a** Frozen brain sections from a CADASIL patient and a healthy control containing an arteriole (left panels) or a capillary (right panels) were stained for LTBP-1 and analyzed by fluorescence microscopy. **b** Frozen brain sections of three CADASIL patients were stained for LTBP-1 and Notch3-ECD and analyzed by confocal fluorescence microscopy. **c** LTBP-1 co-fractionates with Notch3-ECD. The final (β-ME-containing) fractions of sequentially extracted brain vessels from CADASIL patients and controls were immunoblotted for Notch3-ECD, LTBP-1 and β-tubulin. Shown is a representative blot from two independent experiments. (CAD: CADASIL patient; Ctrl: control).

Members of the latent TGF-β binding protein (LTBP) family, in particular LTBP-1, are key regulators of TGF-β bioavailability within the ECM [38] and were found to co-fractionate with Notch3-ECD deposits [30]. We selected LTBP-1, the most prominent member of this family, for analysis and performed immunofluorescence staining on frozen brain sections. In control samples a weak immunoreactivity in brain arterioles and capillaries was observed (Figure 3a). In contrast, staining was markedly increased in CADASIL vessels and moreover restricted to granules reminiscent of Notch3-ECD deposits, as most clearly seen in capillaries. Indeed, co-staining with Notch3-ECD yielded almost completely overlapping signals in all patients examined (Figure 3b) suggesting specific LTBP-1 recruitment into Notch3-ECD deposits. To verify this finding by an alternative method, we performed biochemical enrichment of Notch3-ECD from isolated brain vessels using sequential protein extraction based on a protocol established previously [30]. Immunoblotting of the final (β-ME-containing) fraction for Notch3-ECD confirmed its enrichment in CADASIL but not control vessels (Figure 3c). Importantly, LTBP-1 showed a similar fractionation behavior further suggesting an association with Notch3-ECD (Figure 3c).

### Latency-associated peptide (LAP) accumulates in the tunica media of CADASIL vessels

**Figure 4:**
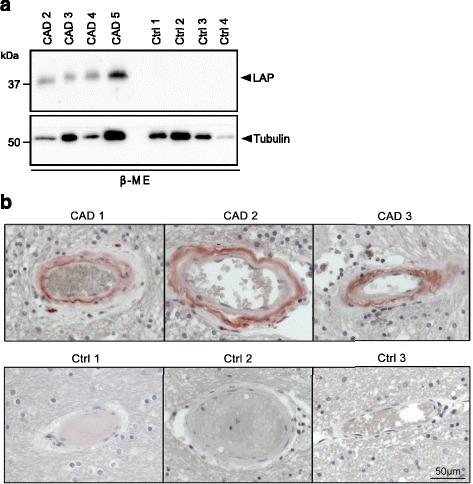
**LAP accumulates in CADASIL brain vessels. a** LAP co-fractionates with Notch3-ECD. The β-ME fractions of sequentially extracted brain vessels from CADASIL patients and controls were immunoblotted for LAP and β-tubulin. Shown is a representative image of two independent extractions. **b** LAP accumulates in the tuncia media of CADASIL patient vessels. Paraffin-embedded brain sections were stained for LAP and counterstained with hematoxylin. (CAD: CADASIL patient; Ctrl: control).

The major biological function of LTBP-1 is the sequestration of latent TGF-β within the ECM by covalent interaction with latency-associated peptide (LAP) [38,39]. We therefore analyzed LAP levels by immunoblotting of the β-ME fraction of purified brain vessels (Figure 4a) as well as by immunohistochemical staining of paraffin sections (Figure 4b) and detected a clear accumulation in patient, but not control samples. Most importantly, LAP immunoreactivity was restricted to the tunica media (Figure 4b), the vessel layer containing Notch3-ECD deposits. Due to the lack of a suitable antibody, co-localization studies could not be performed. Nonetheless, our findings suggest an LTBP-1-mediated sequestration of LAP into Notch3-ECD aggregates and point to an involvement of the TGF-β pathway in CADASIL pathogenesis.

### LTBP-1 directly interacts with Notch3-ECD in vitro via its N-terminus

**Figure 5:**
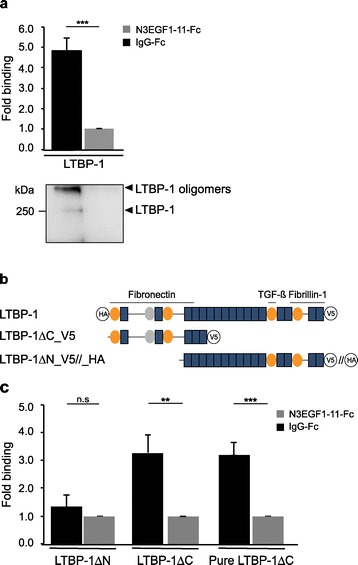
**LTBP-1 binds to immobilized Notch3 in a solid-phase binding assay. a** The interaction of full-length LTBP-1 derived from conditioned cell supernatants with an immobilized Notch3 fragment (N3EGF1-11-Fc) is increased 4.9-fold when compared to a control ligand (IgG-Fc). Results are expressed as mean + SEM of seven independent experiments. Bound LTBP-1 was re-evaluated by immunoblotting and predominantly detected in oligomeric form. **b** Schematic representation of the used LTBP-1 constructs and their domain organization including cysteine-rich repeats (orange ovals), a hybrid domain (grey ovals), EGF-like repeats (blue boxes) and V5-His or HA tags (black circles). The fibronectin, TGF-β, and fibrillin-1 binding regions are indicated. Note that LTBP-ΔC-HA was used in assays with cell supernatants and LTBP-ΔC-V5 in assays requiring purified LTBP-1. **c** LTBP-1 binding to Notch3 is mediated by its N-terminus. While the N-terminal deletion variant LTBP-1ΔN does not bind significantly, the C-terminal deletion variant LTBP-1ΔC, either from conditioned supernatant or in purified form, shows significant interaction. Results are expressed as mean + SEM of five (LTBP-1ΔC_V5) and four (LTBP-1ΔN_HA) independent experiments. n.s.: not significant, ***p < 0.001; **p < 0.01; Mann–Whitney Test.

**Figure 6:**
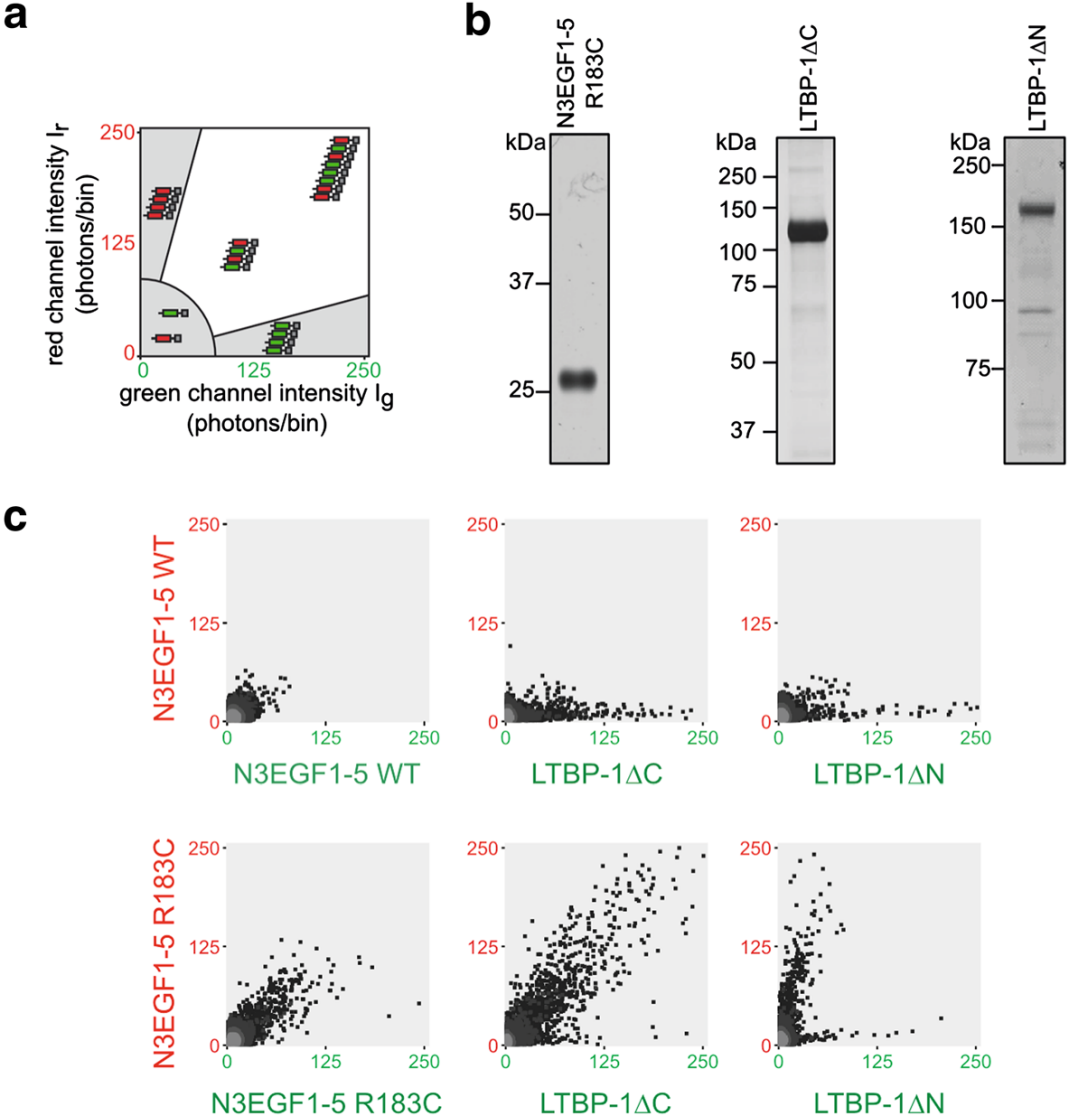
**LTBP-1ΔC specifically co-aggregates with mutant Notch3. a** Notch3-ECD multimer formation is monitored by SIFT and data illustrated in a 2D histogram: axes represent the intensity of photons per bin in the detector channel (green channel along the x-axis, red channel along the y-axis). While monomers and homomeric multimers result in data points in the lower left histogram area and along the axes respectively, heteromeric multimers are represented as high-intensity, dual-color signals in the white sector. **b** Purity of proteins used in the aggregation assay. Shown are silver-stained gels containing the elution fractions after metal-ion matrix (LTBP-1ΔC or LTBP-1ΔN) or Halo-tag-mediated purification (N3EGF1-5 R183C). **c** SIFT data from different protein combinations: while no high-molecular-weight particles are detected with N3EGF1-5 WT, typical aggregates are formed by N3EGF1-5 R183C and when combined with LTBP-1ΔC. In all other combinations homomeric multimers are detected indicating self-aggregation. Shown are representative images of 2–5 experiments. WT: wild-type.

Since recruitment of ECM proteins into Notch3-ECD deposits might be facilitated by direct protein-protein interactions [30], we investigated binding of LTBP-1 to Notch3-ECD by applying a solid-phase binding assay, an approach successfully used in the past to analyze interaction partners of Notch3 [29] or LTBP-1 [40]. Due to the difficulty to purify correctly folded full-length Notch3-ECD [28], a recombinantly generated Notch3-ECD fragment comprising EGF-like repeats 1–11 and an IgG-Fc affinity tag (N3EGF1-11-Fc) was used. Equally efficient immobilization of N3EGF1-11-Fc and the control ligand IgG-Fc to microtiter plate wells was demonstrated by Western Blotting (Additional file 1: Figure S1). LTBP-1 was provided in soluble form by using conditioned supernatants of transfected HEK293T cells. The amount of LTBP-1 bound to N3EGF1-11-Fc was almost 5-fold increased in comparison to the control ligand (Figure 5a). This was verified by SDS-PAGE and immunoblotting of the material recovered from microtiter plates after assay completion revealing a prominent high-molecular-weight band most likely representing oligomeric LTBP-1 not dissolved under the solubilization conditions used (Figure 5a). These findings suggested a direct interaction between LTBP-1 and the first 11 EGF-like repeats of the Notch3-ECD. To map the binding domain within LTBP-1, two deletion constructs lacking either the N-terminus with the fibronectin interaction domain (LTBP-1ΔN) or the C-terminus containing the LAP binding site (LTBP-1ΔC) were generated (Figure 5b). Analysis of conditioned supernatants from HEK293T cells overexpressing either one of these constructs revealed significant binding only for LTBP-1ΔC (Figure 5c). To exclude an indirect interaction mediated by other factors present in the supernatant, the binding assay was performed with LTBP-1ΔC affinity-purified via its C-terminal His tag (see Figure 6b) and revealed a binding capacity similar to LTBP-1ΔC-containing supernatant (Figure 5c). This finding suggested that the sequestration of LTBP-1 into Notch3-ECD deposits is mediated by a direct interaction involving its N-terminal region.

### LTBP-1 co-aggregates with mutant Notch3

To provide proof for a direct participation of LTBP-1 in the Notch3-ECD aggregation process, a previously established *in vitro* assay recapitulating the multimerization of mutant Notch3 was used [27,28]. It is based on scanning for intensely fluorescent targets (SIFT), a unique technique allowing the quantitation of *de novo* protein aggregation in solution [26]. Purified proteins are labeled with different fluorescent dyes and pairwise incubated to allow the formation of multimeric particles whose size and relative composition is quantified by SIFT and presented in a two-dimensional plot as depicted in Figure 6a. While monomers and low-molecular-weight oligomers are shown as low-intensity signals, homomeric and heteromeric multimers are represented as mono-colored or dual-colored high-intensity signals, respectively.

For our analyses we used the previously described wild-type and R183C mutant-carrying Notch3 fragments encompassing EGF-like repeats 1-5 (N3EGF1-5) [28] as well as the LTBP-1 deletion variants LTBP-1ΔN and LTBP-1ΔC. Figure 6b shows the elution fractions of the respective purification procedures used for fluorescent labeling (purity of N3EGF1-5 wild-type was comparable to the R183C mutant, data not shown). In line with our published data, N3EGF1-5 R183C formed higher-order dual-color multimers, while the corresponding wild-type fragment did not show this tendency (Figure 6c). When LTBP-1ΔC or LTBP-1ΔN were analyzed in combination with wild-type N3EGF1-5, only single-color LTBP-1 multimers were detected indicating self-aggregation, a finding already observed in the solid-phase binding assay (see Figure 5a). Predominantly Notch3-containing multimers were obtained when N3EGF1-5 R183C was combined with LTBP-1ΔN lacking the Notch3 interaction domain (Figure 6c). In contrast, the combination of N3EGF1-5 R183C and LTBP-1ΔC yielded dual-color multimers with a distribution typical for mutant Notch3 fragments suggesting the generation of aggregates containing both proteins. In summary, these findings provide further support for our hypothesis that LTBP-1 is a component of Notch3-ECD deposits.

## Discussion

The accumulation and deposition of Notch3-ECD, an early manifestation and hallmark of CADASIL [16], is considered the starting point of a chain of pathological events eventually causing brain vessel dysfunction [9]. Motivated by recent evidence implicating the TGF-β pathway in sporadic and familial SVD [32,33] and by the identification of fibronectin, fibrillin-1 and members of the LTBP family in brain fractions enriched for Notch3 deposits [30], we studied the role of these ECM constituents in CADASIL-related Notch3-ECD aggregation. For fibronectin and fibrillin-1, fibril-forming glycoproteins required for the LTBP-mediated sequestration of latent TGF-β [35], we observe an enrichment in the tunica intima and tunica adventitia of brain vessels from CADASIL patients. A similar increase in fibronectin has previously been reported in a single CADASIL case [41]. However, we do not detect co-localization with Notch3-ECD deposits in the tunica media. This finding argues against a direct involvement of fibronectin and fibrillin-1 in Notch3-ECD aggregation, but rather suggests that their accumulation is due to a fibrotic process secondary to Notch3-ECD deposition as previously reported for various types of collagens [5,6].

In contrast, for LTBP-1, the most prominent member of the LTBP family of TGF-β signaling regulators [38], we find both an accumulation and a striking co-localization with Notch3-ECD deposits. The presence of this finding in three unrelated patients carrying different *NOTCH3* mutations argued for its specificity and prompted us to investigate the relationship between LTBP-1 and Notch3-ECD in more detail. By applying a solid-phase binding assay we detected a previously unknown direct interaction between the LTBP-1 N-terminal domain and the first 11 EGF-like repeats of Notch3-ECD. This assay relied on the efficient immobilization of the Notch3 protein which could not be achieved with our Notch3-ECD fragments encompassing EGF repeats 1–5 preventing a more precise mapping of the binding site. Likewise, the effect of CADASIL mutations on the LTBP-1-Notch3 interaction could not be investigated since a mutant EGF1-11 fragment was not available. Therefore, mutant-specific effects were studied in a previously established Notch3 aggregation assay [27,28]. While the LTBP-1 deletion variant containing the Notch3 binding site showed strong co-aggregation with the archetypal Notch3 mutant R183C, this tendency was not observed with the variant lacking the Notch3 interaction domain. Altogether, these results strongly suggest the recruitment of LTBP-1 into Notch3-ECD deposits by a co-aggregation mechanism requiring a direct interaction.

Our observations extend previous results implicating TIMP-3 and vitronectin in Notch3-ECD deposit formation [30]. In this study, also LTBP-2 and LTBP-4 had been identified by mass spectrometry in human and murine Notch3-ECD preparations, respectively, supporting our data on LTBP-1. The failure to detect it by the proteomic approach might be due to the fact that, instead of isolated vessels, whole brain tissue was used possibly containing minute amounts of LTBP-1. Alternatively, it might be generally difficult to detect LTBPs by mass spectrometry, a possibility supported by the low number of specific peptides assigned to LTBP-2 and LTBP-4 [30]. LTBP-2 does not bind to TGF-β [42] and was thus not investigated in our study. We attempted to analyze LTBP-4 in our autopsy material and observed a tendency towards enrichment in patient samples by immunoblotting of β-ME fractions. However, immunohistological analyses could not be interpreted due to unclear antibody specificity. Thus, further studies are required to assess the role of the individual LTBP family members in the Notch3-ECD aggregation process.

TGF-β, a well-described regulator of blood vessel formation and homeostasis [43], is secreted from cells as inactive complex consisting of the mature ligand, the TGF-β prodomain (also called latent TGF-β associated peptide, LAP) and a member of the LTBP family. Sequestration within the ECM is mediated by covalent interactions between LTBPs, fibronectin and fibrillins resulting in the generation of a reservoir of inactive TGF-β, from which the mature ligand can be released by a variety of mechanisms including proteolysis [35,38]. In agreement with this, we find increased levels of LAP in the tunica media of CADASIL vessels indicating a dysregulation of TGF-β signaling. Although co-localization studies with Notch3-ECD could not be performed, our data provide evidence for an involvement of the TGF-β pathway in CADASIL pathogenesis.

Dysregulated TGF-β signaling has been demonstrated in a variety of inherited diseases of the vasculature including Marfan syndrome, an autosomal dominant systemic connective tissue disorder primarily associated with manifestations in the cardiovascular, skeletal and ocular systems [44]. It is caused by mutations in fibrillin-1 which lead to compromised microfibril function, reduced TGF-β sequestration and increased TGF-β signaling activity. Elevated TGF-β levels have also been reported in CARASIL, a rare recessively inherited form of SVD sharing several features with CADASIL [45]. It is caused by loss-of function mutations in the gene encoding high temperature requirement protein A1 (HtrA1), a conserved serine protease with a putative role in the TGF-β pathway [33]. CARASIL mutations have been reported to prevent processing of pathway components and to result in elevated TGF-β signaling. Thus, it is tempting to speculate that a dysregulation of TGF-β activity might represent a common feature of both CADASIL and CARASIL. More detailed analyses of the different aspects of the TGF-β pathway in both diseases are required to substantiate its role in inherited SVD.

## Additional file

Additional file 1: Figure S1. N3EGF1-11-Fc and IgG-Fc display similar immobilization efficiency. Bound proteins were recovered from wells by the addition of Laemmli buffer after assay completion and compared to 100 ng purified protein. N3EGF1-11-Fc and IgG-Fc were immunodetected using an anti-human-HRP antibody.
