# Supplementary figures and images for: Sequestration of latent TGF-β binding protein 1 into CADASIL-related Notch3-ECD deposits
Source: Acta Neuropathol Commun. 2014 Aug 13;2:96. doi: 10.1186/s40478-014-0096-8 (PMC4243959; doi:10.1186/s40478-014-0096-8)

## Additional file 1 – Figure S1

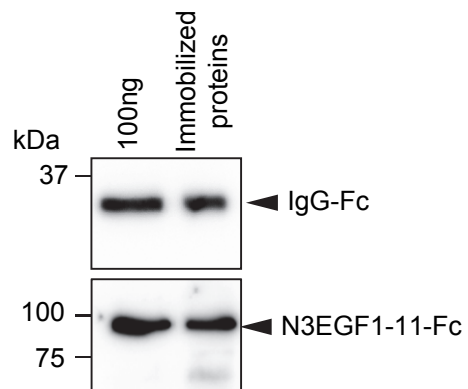

Supplement: Additional file 1: Figure S1. — N3EGF1-11-Fc and IgG-Fc display similar immobilization efficiency. Bound proteins were recovered from wells by the addition of Laemmli buffer after assay completion and compared to 100 ng purified protein. N3EGF1-11-Fc and IgG-Fc were immunodetected using an anti-human-HRP antibody. [file 40478_2014_96_MOESM1_ESM.pdf]
